# Low Habitual Dietary Calcium and Linear Growth from Adolescence to Young Adulthood: results from the China Health and Nutrition Survey

**DOI:** 10.1038/s41598-017-08943-6

**Published:** 2017-08-22

**Authors:** Aiping Fang, Keji Li, He Li, Meihan Guo, Jingjing He, Xin Shen, Jie Song

**Affiliations:** 1 Department of Nutrition, School of Public Health, Sun Yat-sen University, Guangzhou, 510080 China; 2 Department of Nutrition and Food Hygiene, School of Public Health, Peking University, Beijing, 100191 China

## Abstract

Evidences from clinical trials and meta-analyses of calcium supplementation in linear growth have given conflicting results, and few longitudinal studies have investigated the long-term associations between dietary calcium and linear growth, especially in the population with low-calcium plant-based diets. We investigated the prospective associations of low habitual dietary calcium with adult height and height-for-age *z*-score (HAZ) from adolescence to adulthood among 2019 adolescents from the China Health and Nutrition Survey (CHNS). The average dietary calcium intakes were 426(standard deviation: 158) mg/d in boys and 355(134) mg/d in girls during adolescence. During a median follow-up of 7.0 (interquartile range: 5.9–9.0) years, boys reached an average of 169.0(6.7) cm and girls reached 158.4(5.8) cm in adulthood. After adjusting for other potential confounders, non-linear regression found that boys with dietary calcium intakes below 327 mg/d had shorter adult stature, and those taking over 566 mg/d had faster height growth whether adjusting for physical exercises level or not. No significant associations were found in girls. Our study suggests that in boys with plant-based diets, higher dietary calcium intake during adolescence is associated with faster height growth, but not with adult height; calcium intake below 300 mg/d may result in shorter adult stature.

## Introduction

Calcium is necessary for bone growth and skeletal development^1^; hence, it is plausible that adequate calcium intake is considered as a prerequisite for normal linear growth. Calcium deposition into bone reaches maximal accretion during pubertal growth spurt. Adolescents are recommended to take at least 1000–1300 mg of calcium daily for optimal bone health^1–3^. This recommendation level, however, is sparsely met by children with plant-based diets, which is characterized by higher intakes of cereals, legumes, vegetables, and lower quantities of animal foods, for the historical absence of dairy foods, whose dietary calcium intakes are often as low as 300–400 mg/d^4^. Although there are increasing trends in the percentage of milk consumption (from 2.88% in 1991 to 13.88% in 2006) and the average daily intake of milk (from 3.90 g in 1991 to 26.11 g in 2006) among Chinese children and adolescents, the overall intake level remains quite low^5^.

Low calcium intake during growth may lead to growth retardation and suboptimal peak bone mass^6, 7^. However, evidences from clinical trials and meta-analyses of calcium supplementation in linear growth have drawn conflicted results^6, 8–13^, and few longitudinal studies have focused on the long-term association between dietary calcium and linear growth, especially in children with plant-based diets in developing countries.

In this context, we investigated prospective associations of low habitual dietary calcium during adolescence with adult height and height growth using longitudinal growth data from the China Health and Nutrition Survey (CHNS). This cohort provides a unique opportunity to gain insight into the long-term effects of low calcium intake on linear growth, with detailed information on dietary calcium intake, anthropometric data, behavioral, social, and lifestyle confounders during growth until adulthood.

## Methods

### The China Health and Nutrition Survey

This was a longitudinal growth cohort study from the China Health and Nutrition Survey (1989–2011). The CHNS is an ongoing, longitudinal, and prospective household-based cohort study of nine waves (i.e., 1989, 1991, 1993, 1997, 2000, 2004, 2006, 2009 and 2011). Overall response rates were 93% at the individual level and 92% at the household level, based on those who were followed up at least once. The survey procedures were reviewed and approved by the institutional review committees of the University of North Carolina at Chapel Hill (UNC-CH) and the National Institute of Nutrition and Food Safety, China Centre for Disease Control and Prevention (INFS, CCDC), in accordance with the ethical standards laid down in the 1964 Declaration of Helsinki and its later amendments. All respondents provided written informed consents. Cohort profile was described elsewhere^14^.

### Sample and Setting

**Figure 1:**
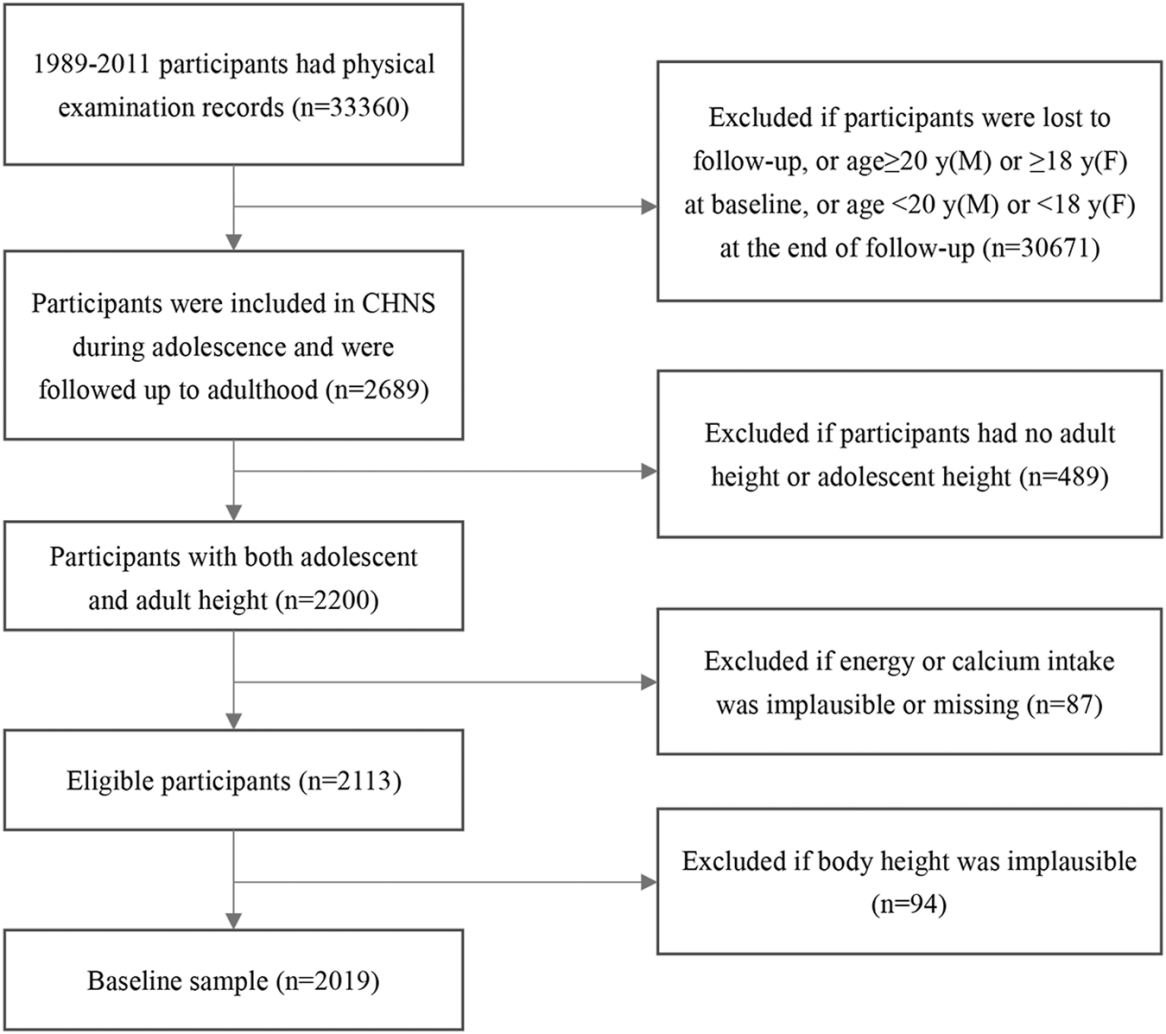
The flow chart of sample selection in the China Health and Nutrition Survey.

All data used in this study were derived from the CHNS 1989–2011. Participants born between 1969 and 1993 were enrolled in the CHNS during adolescence (i.e., 12–19 years of age for boys and 10–17 years of age for girls^15^) and followed up to adulthood. The current analyses included 2019 participants from 226 urban and rural communities in nine provinces in China, after excluding participants with neither adolescent height nor adult height, or with implausible or missing values for height, total energy intake, or dietary calcium (the upper and lower 0.5% of the intakes) (Fig. 1).

### Height measurements

Body heights of both children and their biological parents were measured by trained health workers following standard procedures in each wave^15^. All trained staff passed a competency test to assure intra- and inter-observer reliability. Height without shoes was measured to the nearest 0.1 cm using SECA 206 wall-mounted metal tapes (SECA, Hamburg, Germany).

For children, anthropometric data during growth were recorded from their enrollment in the CHNS and updated in subsequent follow-ups. We took the measured height from the first subsequent survey after the children’s entering adulthood as their adult height, which served as a proxy for cessation of the pubertal growth.

Height-for-age *z*-scores (HAZs) were calculated according to the 2007 World Health Organization (WHO) criteria for boys and girls aged 5 to 19 years or for boys and girls aged 19 years, whichever appropriate^16, 17^.

### Dietary assessment

A combination of 24-hour individual dietary recalls with modification of weighing and measuring household edible oils and condiments consumption for three consecutive days was repeatedly performed to assess individual diet in each survey^18^. All interviewers were trained and passed an examination before collecting data. We calculated nutrient intakes as the nutrient content of a standard portion size of 100 grams multiplied by the consumption of each food item. The China Food Composition Tables (FCTs)^19–22^ were utilized to obtain nutrient data. To better account for long-term dietary intakes during adolescence, we averaged the nutrient intakes from all dietary surveys over puberty, despite that the number of dietary surveys may vary from subject to subject depending on the differences in each individual’s age of entry into the cohort and follow-ups during adolescence. Gender-specific average nutrient intakes during adolescence were further adjusted for mean total energy intake between start and end of adolescence (2561 kilocalories for boys and 2073 kilocalories for girls, respectively) using the residual method^23^ after log transformation.

To learn more about dietary patterns of the participants, we additionally calculated the edible weights of raw food consumed by individuals at baseline and subsequent follow-ups during adolescence and then divided them into 24 food groups (i.e., cereals, tubers, starches, soybean, mixed beans, vegetables, fungi and algae, fruit, nuts and seeds, meat, poultry, milk, eggs, seafood, infant foods, ethnic foods, fast foods, beverages, alcohol, sugars and preserves, animal oil, vegetable oil, condiments, and others), according to the classification system of foods adopted by the China FCT 2002 & 2004^21, 22^. Average food consumption by each group during adolescence of an individual, expressed in units of g/1000 kcal/d, was obtained through dividing the average intake of this group from all dietary surveys over puberty by the corresponding average energy intake. The contribution of the above food groups to dietary protein and calcium during adolescence were estimated from average energy adjusted protein and calcium intakes from each food sources and their percentage of the total average intakes over puberty. Similar procedures were used to assess dietary protein and calcium sourced from plant foods (i.e., cereals, tubers, starches, soybean, mixed beans, vegetables, algae, fruit, nuts and seeds, and vegetable oil), animal foods (i.e., meat, poultry, milk, eggs, seafood, and animal oil) and other foods (i.e., fungi, beverages, alcohol, sugars and preserves, and condiments). Infant foods, ethnic foods, fast foods, and others were assigned to one of the three categories based on their food raw materials.

### Additional information

Structured questionnaires were used to collect demographic and lifestyle information. In this study, we only considered variables directly relevant to the research purposes, including children’ gender, age, year of birth, information on stratum and physical exercises. Physical exercises data on the frequency and mean duration of sport participation have been collected from children since 1997, and physical exercises level was recorded as less than 2 hours/week, 2–5 hours/week and more than 5 hours/week in each around. The height of children’ biological parents, obtained from repeated physical measurements, was averaged to minimize intra-individual variation. The urbanization index, a time-varying continuous variable, was constructed to reflect economic, demographic, social welfare, and infrastructural diversity at the community level^24^. Latitude and longitude information of provincial capitals were obtained from Google earth (http://www.earth.google.com/) to locate geographic positions of the survey sites. We additionally extracted annual average direct normal radiation (DNR) from July 1983 to June 2005 from the NASA Surface Meteorology and Solar Energy (https://eosweb.larc.nasa.gov/cgi-bin/sse/grid.cgi?email=skip@larc.nasa.gov) to estimate ultraviolet radiation of all parts. Weight in light, indoor clothing was measured to the nearest 0.1 kg using SECA 880 scales (SECA, Hamburg, Germany). Body mass index (BMI) was calculated as body weight in kg/(height in m^2^).

### Statistical Analysis

For continuous variables, means and standard deviations (SDs/SEs) were reported in normal distribution, and medians and interquartile ranges (IQRs) were estimated in skewed distribution. Categorical variables were expressed as numbers and percentages (%). We used two-independent samples t tests, two-independent nonparametric tests, or chi-square tests as appropriate to compare the baseline characteristics before and after excluding ineligible participants. The variation of nutrient intakes during growth was detected by using linear regression models of energy and energy adjusted nutrient intakes and participants’ age during adolescence in each survey around.

In order to assess the contribution of dietary calcium intake during adolescence to adult height and to eliminate the effects of dietary protein intake over puberty and other factors on the associations between dietary calcium and adult height, we performed multiple linear regression models of adult height with or without average energy adjusted dietary calcium intake as an independent variable, respectively, and other potential confounders included baseline age, height, and weight, adult age and weight, year of birth, paternal and maternal height, urbanization index, latitude, DNR, average dietary intake of total energy, average energy adjusted dietary intakes of protein, vitamin A, iron, and zinc during adolescence (all continuous), stratum, and household income level (all categorical). Non-linear trends of adult height were additionally analyzed using restricted cubic spline (RCS) functions, with four knots located at percentiles 5th, 35th, 65th, and 95th of the average energy adjusted dietary calcium intake during adolescence. The reference level was set to 400 mg of dietary calcium, corresponding to the average daily intake for Chinese residents^4^. In order to estimate the contribution of dietary calcium to height growth velocity, a similar analysis was performed to investigate the curvilinear dose-response association between HAZ and dietary calcium during growth using linear generalized estimating equations (GEE) models. All the RCS analyses were conducted using the RCS_Reg SAS Macro provided by Desquilbet and Mariotti^25^. Given that the growth pattern of stature is S-shaped from childhood to adulthood^26^, we treated children’ age as RCS function as well. The models were adjusted for other potential covariates as well. Product interaction terms were added to investigate effect measure modification between age and dietary calcium, between age and dietary protein, and between dietary calcium and protein in multivariable models, which were found not significant (*P* > 0.05) and thus not included in the results. Since 1221 children (60.5%) were enrolled during 1989–1993 and lacked baseline physical exercises information in this study, we re-ran our main analyses with additional adjustment for physical exercises level by utilizing subgroup data after 1997. Missing values for covariates (e.g., weight, paternal and maternal height) were imputed by Markov chain Monte Carlo multiple imputation method. Sensitivity analysis with restriction to complete data did not alter our interpretation of the results. All analyses were conducted for boys and girls separately.

The statistical analyses were performed with SAS version 9.2 (SAS Institute, Cary, NC, USA). Graphs were generated using Microsoft Excel software version 2016 (Microsoft, Redmond, WA, USA). A two-sided *P* value of less than 0.05 was considered as the significant level for all analyses.

## Results

A comparison of the growth cohort before and after excluding ineligible participants, showed no significant difference in baseline characteristics (*P* > 0.05) (Supplemental Table 1). Overall characteristics of the youth cohort used in this study are presented in Table 1. Of the 2019 participants (1165 boys and 854 girls) included in the analyses, 1221 (60.5%) were included in the cohort before 1993, 679 (33.6%) were included between 1997 and 2000, and 119 (5.9%) were included after 2004. During a median follow-up of 7.0 (IQR: 5.9–9.0) years and 15056.3 person years in total, the median gains in height were 8.8 (IQR: 1.9–20.2) cm for boys and 9.0 (IQR: 3.0–19.5) cm for girls from baseline to adulthood, and boys reached an average of 169.0(6.7) cm and girls reached 158.4(5.8) cm in adulthood.

**Table 1:** Characteristics of adolescent boys and girls with plant-based diet.

	Boys (*n* = 1165)	Girls (*n* = 854)
**At baseline** ^**a**^		
Age (years)	15.4(2.3)	13.5(2.3)
Height (cm)	157.3(12.0)	146.7(10.8)
Weight (kg)	47.0(11.4)	39.1(9.4)
BMI (kg/m^2^)	18.7(2.6)	17.9(2.7)
HAZ	−1.24(1.13)	−1.18(1.11)
**End of follow-up** ^**a**^		
Age (years)	23.2(3.0)	20.4(2.4)
Height (cm)	169.0(6.7)	158.4(5.8)
Weight (kg)	61.5(10.3)	51.6(6.6)
BMI (kg/m^2^)	21.5(2.9)	20.5(2.3)
HAZ	−1.03(0.92)	−0.73(0.88)
**Paternal height (cm)** ^a,d^	165.2(6.3)	165.6(6.4)
**Maternal height (cm)** ^a,d^	154.6(5.9)	154.9(6.0)
**Daily dietary intake** ^**e**^		
Total energy (kcal)^a^	2561(602)	2073(460)
Protein (g)^a^	76.8(12.9)	62.9(10.3)
Vitamin A (ugRE)^b^	387(208,623)	330(178,528)
Calcium (mg)^a^	426(158)	355(134)
Iron (mg)^a^	24.1(9.7)	19.9(6.2)
Zinc (mg)^a^	12.4(2.0)	10.0(1.8)
**Urbanization index** ^a,g^	47.9(17.7)	49.8(17.9)
**Latitude** ^a^	31.3(6.2)	32.2(6.3)
**DNR (kWh/m** ^**2**^ **/d)** ^**a**^	3.47(1.06)	3.60(1.07)
**Year of birth** ^b^	1979(1975,1984)	1981(1977,1986)
**Wave at entry** ^**c**^		
1989–1993	709(60.9)	512(60.0)
1997–2000	390(33.5)	289(33.8)
2004–2009	66(5.7)	53(6.2)
**Stratum** ^**c**^		
Rural	908(77.9)	639(74.8)
Urban	257(22.1)	215(25.2)
**Physical exercises level** ^**c**,**f**,**g**^		
<2 hours/week	108(32.0)	150(53.0)
2~5 hours/week	92(27.2)	97(34.3)
≥ 5 hours/week	138(40.8)	36(12.7)
**Household income level** ^**c**,**g**^		
Low (<¥2000)	408(35.0)	319(37.4)
Medium (¥2000–5000)	506(43.4)	360(42.2)
High (≥¥5000)	251(21.5)	175(20.5)

^a^Data shown is mean (SD). ^b^Data shown is median (IQR). ^c^Data shown is *n* (%). ^d^Average height between the baseline and the follow-ups. ^e^Energy adjusted average nutrient intakes during adolescence were estimated from a mean number of 1.8 three-day 24-hour dietary recalls (range: 1–3) obtained during age 12–19 in boys and 10–17 years in girls using the residual method after log transformation. ^f^Data was only collected in and after 1997. ^g^Information was only presented in the baseline. BMI: body mass index; HAZ: height-for-age *z*-score; DNR: annual average direct normal radiation; SD: standard deviation; IQR: interquartile range.

**Figure 2:**
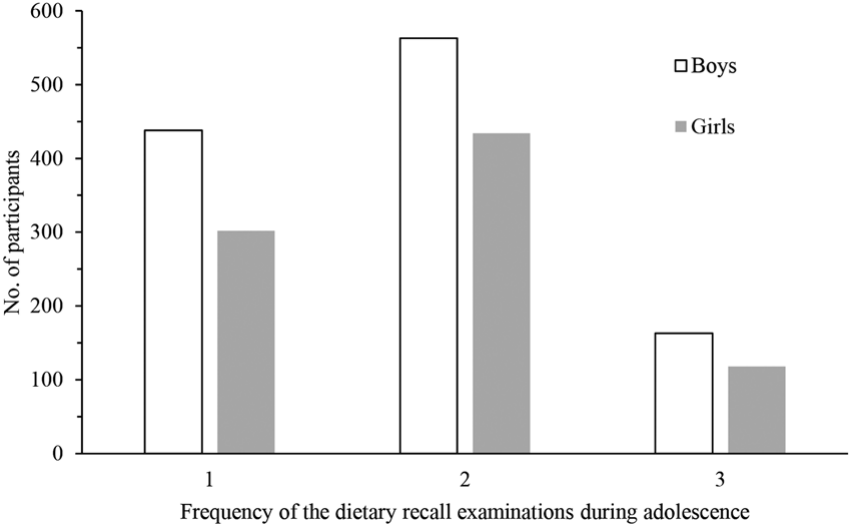
Distribution of the repeated three-day 24-hour dietary recall examinations during adolescence in boys and girls with plant-based diet.

The mean number of the repeated dietary measurements was 1.8 (range: 1–3) obtained during age 12–19 in boys and 10–17 years in girls in the cohort (Fig. 2). Cereals (196.0 vs. 191.8 g/1000 kcal/d) and vegetables (122.6 vs. 129.3 g/1000 kcal/d) were the most popular foods consumed by boys and girls during adolescence, however, a much lower consumption of animal foods was observed with average intakes of meat, seafood, poultry and eggs of 39.6 g/1000 kcal in boys and of 41.8 g/1000 kcal in girls per day. Average daily milk intakes during adolescence were only 2.8 g/1000 kcal for boys and 3.3 g/1000 kcal for girls, respectively (Supplemental Table S2). The average percentage of dietary protein from plant foods was 80.2% for boys and 79.3% for girls, and the average percentage of dietary calcium from plant foods was 92.0% for boys and 91.4% for girls during adolescence (Supplemental Table S3). 95.5% of boys and 94.3% of girls took at least 50% of dietary protein from plant foods, and 98.5% of boys and 98.1% of girls took at least 50% of dietary calcium from plant foods (Supplemental Table S4). Total energy intakes increased with participants’ age over puberty (*P* < 0.001), while other nutrient intakes (protein, vitamin A, calcium, iron and zinc) remained stable after adjusting for total energy during adolescence in both boys and girls (Supplemental Table S5). The average energy adjusted intakes of dietary calcium were 426(158) mg/d in boys and 355(134) mg/d in girls, and the average energy adjusted intakes of dietary protein were 76.8 (12.9) g/d in boys and 62.9 (10.3) g/d in girls during adolescence, respectively (Table 1).

As shown in Table 2, after adjusting for other potential confounders, positive linear associations of average energy adjusted dietary intakes of protein and calcium during adolescence with adult height were observed in boys by using the multiple linear regression models, whether to additionally adjust for physical exercises level or not (*P* < 0.05). There were no similar and significant trends in girls (*P* > 0.05). The 95% confidence intervals (CIs) for average dietary calcium intakes during adolescence were 234–732 mg/d for boys and 193–618 mg/d for girls in these models.

**Table 2:** Multivariable adjusted regression analysis of adult height as outcome variable and dietary protein (per 10 g increase) and calcium (per 100 mg increase) as predictors in adolescent boys and girls with plant-based diet.

Outcome: adult height	Boys				Girls			
	*β*	*SE*	*P*	*adj*.*R* ^2^	*β*	*SE*	*P*	*adj*.*R* ^2^
Model 1 Protein intake	0.29	0.12	0.020	0.657	−0.15	0.16	0.362	0.595
Model 2 Calcium intake	0.18	0.08	0.028	0.670	−0.12	0.10	0.241	0.616
Model 3 Protein intake	0.48	0.20	0.014	0.650	−0.25	0.24	0.309	0.570
Model 4 Calcium intake	0.30	0.14	0.038	0.654	−0.03	0.17	0.859	0.567

Model 1 used full data from 1989 to 2011, adjusted for baseline age, height, and weight, adult age and weight, year of birth, paternal and maternal height, urbanization index, latitude, DNR, average dietary intake of total energy, average energy adjusted dietary intakes of vitamin A, iron, and zinc during adolescence, stratum, and household income level. Model 2 adjusted as Model 1 and additionally adjusted for protein intake. Model 3 only used data since 1997, adjusted as model 1, and additionally adjusted for physical exercises level. Model 4 adjusted as Model 3 and additionally adjusted for protein intake. BMI: body mass index; DNR: annual average direct normal radiation; SE: standard error for β.

**Figure 3:**
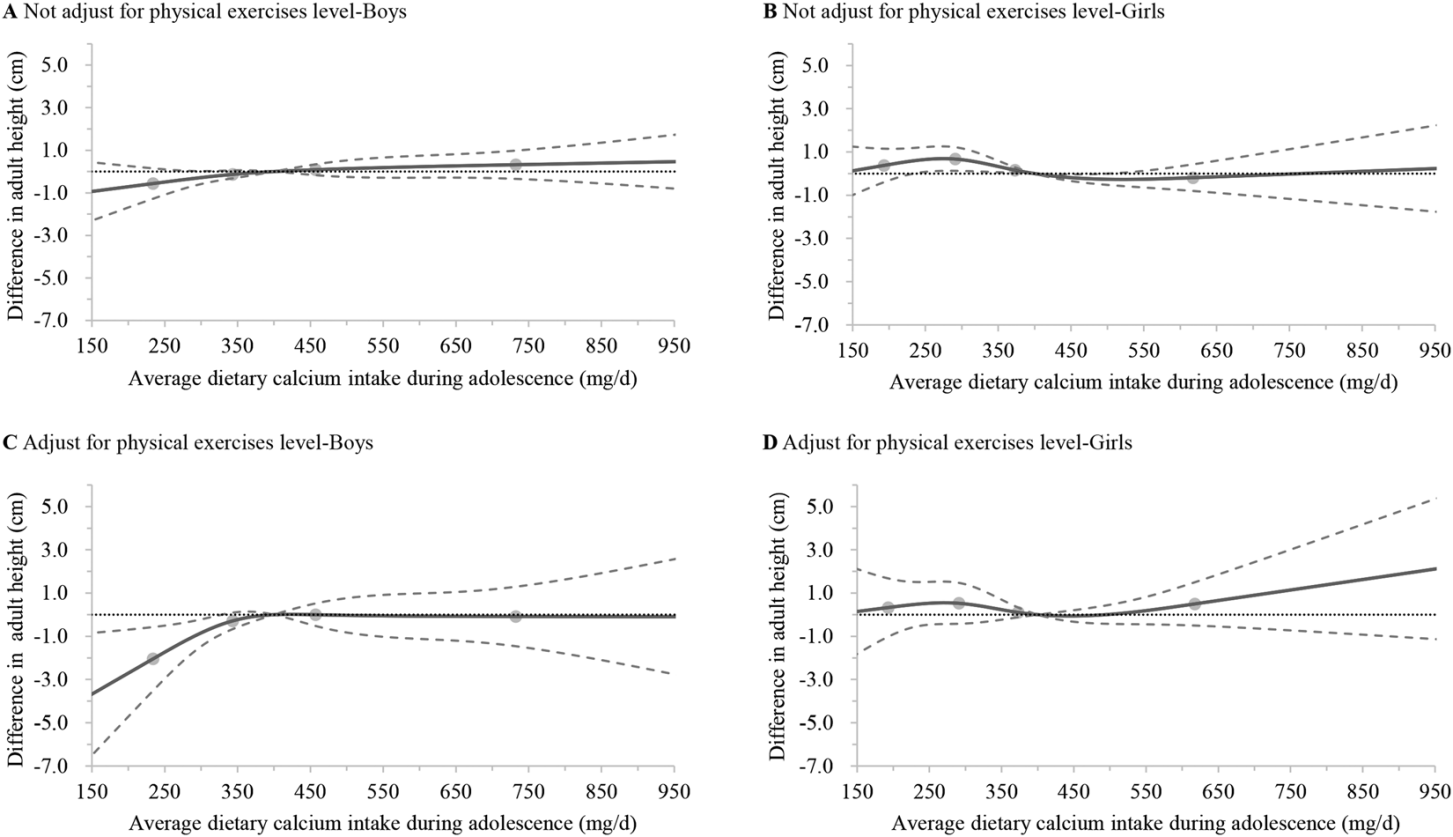
Multivariable adjusted dose-response associations between average dietary calcium intake during adolescence and adult height in boys and girls with plant-based diet. Multivariable adjusted dose-response curves were indicated by solid line and 95% confidence interval by dashed lines. Calcium intake was coded using an RCS function with four knots, represented by dots, located at the 5th, 35th, 65th, and 95th percentiles of the distribution of average intake of dietary calcium. Y-axis represents the difference in adult height between individuals with any value of calcium intake with individuals with 400 mg/d of calcium intake. Model A and B, using full data from 1989 to 2011, were adjusted for baseline age, height, and weight, adult age and weight, year of birth, paternal and maternal height, urbanization index, latitude, DNR, average dietary intake of total energy, average energy adjusted dietary intakes of protein, vitamin A, iron, and zinc during adolescence, stratum, and household income level. Model C and D, only using data since 1997, and additionally adjusted for baseline physical exercises level. RCS: restricted cubic spline; DNR: annual average direct normal radiation.

The dose-response association between average dietary calcium intake during puberty and adult height is further illustrated by the multivariable adjusted spline curves in Fig. 2. Adult height increased rapidly with dietary calcium intake during adolescence and reached a plateau at an intake level of around 400 mg/d in boys (overall association: χ^2^ = 4.76, *P* = 0.1900, and χ^2^ = 8.07, *P* = 0.0445, respectively) (Fig. 3A and C). After additionally adjusting for physical exercises level, we found a threshold dietary calcium intake of about 400 mg/d, and boys whose intakes fell below 327 mg/d during puberty were significantly shorter than those with intakes above this level (difference in adult height −0.47 cm, 95% CI −0.93 to −0.01 cm) (Fig. 3C). However, no more gains in adult height were detected when daily dietary calcium intakes were more than 400 mg. The threshold effects were not significant in girls (overall association: χ^2^ = 6.44, *P* = 0.0920, and χ^2^ = 2.07, *P* = 0.5590, respectively) (Fig. 3B and D).

**Figure 4:**
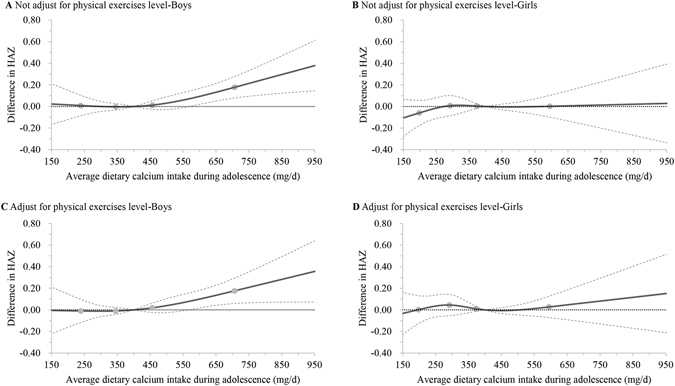
Multivariable adjusted dose-response associations between average dietary calcium intake during adolescence and HAZ from adolescence to young adulthood in boys and girls with plant-based diet. Multivariable adjusted dose-response curves were indicated by solid line and 95% confidence interval by dashed lines. Calcium intake were coded using an RCS function with four knots, represented by dots, located at the 5th, 35th, 65th, and 95th percentiles of the distribution of average intake of dietary calcium. Y-axis represents the difference in height-for-age *z*-score between individuals with any value of calcium intake with individuals with 400 mg/d of calcium intake. Model A and B, using full data from 1989 to 2011, were adjusted for age, weight, year of birth, paternal and maternal height, urbanization index, latitude, DNR, average dietary intake of total energy, average energy adjusted dietary intakes of protein, vitamin A, iron, and zinc during adolescence, stratum, and household income level. Model C and D, only using data since 1997, and additionally adjusted for physical exercises level during growth. HAZ: height-for-age *z*-score; RCS: restricted cubic spline; DNR: annual average direct normal radiation.

In contrast, J-shaped associations between HAZ and average dietary calcium intake during adolescence were observed with the inflexion at an intake level of around 400 mg/d in boys (overall association: χ^2^ = 13.04, *P* = 0.0046, and χ^2^ = 9.57, *P* = 0.0226, respectively) (Fig. 4A and C). Especially, boys who consumed dietary calcium of more than 566 mg/d in adolescence had significantly higher HAZ during growth (difference in HAZ: 0.07, 95% CI: 0–0.14, and 0.08, 95% CI 0–0.15, while not adjusting and adjusting for physical exercises level, respectively). Similar trends were found in girls, but not significant (overall association: χ^2^ = 1.49, *P* = 0.6838, and χ^2^ = 1.18, *P* = 0.7579, respectively) (Fig. 4B and D).

## Discussion

### Principal findings

Chinese boys and girls consumed predominantly plant-based diets during adolescence, regardless of food sources of dietary protein or calcium. This study shows that higher dietary calcium intakes during adolescence is associated with faster height growth in boys, but not with adult height if their dietary calcium intakes are not lower than 400 mg/d; however, very low intakes of dietary calcium (below 327 mg/d) may result in lower adult stature. There is no more benefit on adult height to ingest more calcium once a certain amount has been fulfilled. Height grows slower in the short term in boys with lower calcium intake, but finally catches up with those with higher calcium intake in adulthood, provided that the intake reaches an adequate level of 400 mg/d. This intake is only one half of that for the Westerners to maintain normal linear growth. There are no similar threshold associations in girls.

### Strengths and weaknesses of the study

To our knowledge, this is the first prospective and longitudinal cohort study focusing on associations between low habitual intake of dietary calcium and linear growth in adolescents with plant-based diet. Compared with previous trials, we followed up a larger number of adolescents until adulthood under daily routines, which allowed us to investigate the long-term effects of low habitual calcium intake on linear growth.

Nevertheless, there are some limitations in our study. Pubertal growth and development is a rapid and sequential process, and the follow-up frequency of every two to three years in the CHNS was not adequate for capturing growth pattern individually. Moreover, with little information on pubertal status, we could neither further differentiate the contribution of dietary calcium intake among pre-, mid-, and post-puberty to linear growth, nor determine the exact age at height growth cessation.

### Strengths and weaknesses in relation to other studies

Children with long-term avoidance of milk were reported to have low dietary calcium intakes and short stature^27^. In a 13-month randomized clinical trial, British boys taking calcium supplements of 1000 mg/d were taller by 7 mm than those taking placebos^6^. It has also long been debated whether calcium or milk supplementation can promote linear growth in children and adolescents on a lasting basis. One study showed that Chinese girls aged 10 years at baseline, compared with their control counterparts, increased by 0.7–0.8% in height after milk supplementation for two years, but the promoting effect was not sustained 3 years after the cessation of intervention^10^. An earlier study followed up pre-pubertal children with a mean dietary calcium intake of approximately 567 mg/d for 36 months while providing the intervention children with calcium supplementation in the beginning 18 months, and found no difference in percentage height gains at the end of follow-up between the study and control group^11, 28^. Bonjour *et al*.^29–31^ followed up the Swiss girls from pre-puberty to post-menarche for 7.5 years after the end of a 48-week calcium supplementation trial; their results showed a favorable effect of calcium supplements on height growth, especially for girls whose initial calcium intake was below 880 mg/d, nevertheless the effect was not significant at the end of follow-up. Another 10-month intervention study with a 12-month follow-up concluded that calcium supplementation was not associated with greater height increase in healthy children with habitual dietary calcium intake of 800 mg daily^32^. Notably, none of these studies investigated the long-term effect of supplemental calcium on adult height.

Matkovic *et al*.^9^ conducted a 4-year randomized clinical trial among American girls with a mean dietary calcium intake of about 830 mg/d and extended it for an additional 3 years, which concluded no significant difference in height gain between the supplemented and placebo groups at the end of 7-year follow-ups. In a longitudinal study spanning 13 years, Prentice *et al*.^33^ evaluated the lasting effect of 12-month calcium supplementation on the adolescent height growth in 160 Gambian children with very low habitual calcium intakes, and showed that boys using supplements in late childhood had taller stature in mid-puberty, but shorter adult height. In contrast, our study found that boys who consumed higher dietary calcium grew faster during adolescence, but gained similar height in adulthood to their peers consuming less dietary calcium; however, our study found no effects of dietary calcium on linear growth in girls, which is compatible with the studies of Gambian and UK pre-pubertal and adolescent girls^33, 34^.

Additionally, most clinical trials always predetermined a series of dosage groups with a wide range, which might not be proper to investigate exactly the dose-response association between habitual dietary calcium intakes and linear growth; and most statistical models adopted in analysis were based on the assumption of linear correlation between the two variables. For clarifying them, we compared the associations between dietary calcium intakes during adolescence and linear growth by using both linear and non-linear regression models. The significant association in boys between calcium intake and adult height in linear regression models, however, dismissed in non-linear regression models, to which the strong association between low dietary calcium intake below 327 mg/d and adult height made the most contribution. The non-linear models present a more plausible association over the linear models in distinguishing different calcium intake intervals.

### Possible explanations and implications

In this study, the mean height in early adulthood for boys and girls is less than 1.03 SDs and 0.73 SDs, respectively, of the WHO reference^17^, which is referred as a result of different ethnic backgrounds^35^.

Although genetics remains the chief determinant of human growth and adult height^36^, nutrition plays a crucial role in achieving the genetic growth potentials^37^. The present results may reflect the catch-up growth phenomenon in linear growth in adolescent boys with low habitual dietary calcium intake, showing that there are no remaining deficits of adult height when dietary calcium intake is above 400 mg/d during puberty. However, a lower dietary calcium intake may lead to a shorter stature; this is supported by the calcium balance study conducted in Chinese adolescent boys aged 12 to 17 years, which observed a negative calcium balance at intakes less than 300 mg/d^38^. Vatanparast *et al*.^39^ reported that the average calcium accretion was 92–210 mg/d in Canadian boys and girls aged 9–18 years. Chinese adolescents could satisfy the above requirements through increasing calcium absorption efficiency and decreasing calcium excretion in urine and feces with the typically low dietary calcium intake^38^.

Linear growth, characterized by longitudinal bone growth, which occurs via endochondral ossification at the growth plate at the end of long bones, reaches a dramatic growth spurt over puberty and stagnates until epiphyseal fusion in adulthood^40^. Nevertheless, the mechanism responsible for a specific calcium impact on growth or final height has not been entirely understood. Calcium, as an essential bone-forming mineral, also deposits into bone at maximal accretion rate during the pubertal growth spurt^1^. Calcium supply during adolescence failing to satisfy the calcium needs of the body would have adverse effects on skeleton growth and development^1^. Ginty *et al*.^41^ reported that calcium carbonate supplementation elevated plasma insulin-like growth factor I (IGF-I) concentrations in adolescents, and IGF-I would stimulate the activation of the hypothalamic-pituitary-gonadal axis^40^, which resulted in advanced the age of peak height velocity and consequently regulated longitudinal bone growth^33^. In our study, boys with a threshold calcium intake over 566 mg/d had higher HAZ during adolescence, but there were no significant advantages in adult height over those with dietary calcium intakes between 400 and 550 mg/d, which may be the result of advanced the age of peak height velocity. However, whether dietary calcium have a similar physiological action like calcium supplements remains an open question.

Dietary protein is a major nutritional determinant of linear growth^42^, which is supported by our findings of a positive linear association between dietary protein intake during adolescence and adult height in boys, despite that no interaction was found between dietary calcium and protein intake, i.e., calcium and protein intake show a parallel variation. After adjusting for average protein intakes over the growth period, significant associations for calcium remained in boys. Both Gambian and British boys received 1000 mg supplemented calcium daily for a similar period, while the former had a reduced final height and the latter had an increased final height^6, 33^, and different protein intake may be one of the reasons. In our study, average protein intake during adolescence was 76.8 g/d in males and 62.9 g/d in females, respectively, which were quite normal and not particularly low, according to the Chinese Dietary Reference Intakes for protein^2^. Although the diets consumed by Chinese adolescents were primarily plant-based, the dietary protein supply may have satisfied the demands for protein of the body. In contrast, dietary calcium deficiency has always been a critical public health concern for decades, even nowadays in China^4^.

The influence of diet may differ between the genders in linear growth, and boys seem to have greater potentials for skeletal expansion than girls. There is a pronounced sexual dimorphism in cortical porosity of bone between adolescent boys and girls, which may reflect lower intracortical bone remodeling in girls^43–45^. Boys accrete bone predominantly through periosteal apposition, whereas girls increase in fat mass relative to lean mass and bone mass^46^, and accrete bone at endocortical surfaces during growth^45^. In girls, dietary calcium and protein intakes fail to show significant effects on height growth, which might be a result of girls’ relatively small skeletal sizes and more efficient adaptation to suboptimal food supply to support their reproduction^38, 47^, however, it warrants further exploration.

Pubertal growth and development may be affected by dietary factors (e.g., energy, protein, iron, and vitamin A) other than calcium^37^. For better growth, emphasis, therefore, should be placed on improving a comprehensive nutritional status, with an eye on adolescent boys with very low dietary calcium intakes. Given the findings from previous longitudinal studies^9, 11, 31, 33^ that extra calcium intake brings no additional benefits on adult height and that calcium supplementation only transiently enhances stature growth (or even has unintended adverse effects), adolescents who have ingested enough calcium from their plant-based diet may not have to take calcium supplements.

### Remaining questions and future research

The results need to be replicated in larger cohort studies with more frequent follow-ups. Long-term randomized clinical trials in the population with plant-based low-calcium diet are needed to verify whether the adaptation to low calcium intake has gender difference in linear growth.

## Conclusions

In boys with plant-based low-calcium diet, calcium intake below 300 mg/d during adolescence is associated with shorter adult stature, while a dietary calcium intake higher than 400 mg/d will not lead to a taller adult stature; dietary calcium intake over 570 mg/d links to a faster height growth, but not to the absolute adult height;. In girls, habitual calcium intake is not associated with linear growth.

## Electronic supplementary material

Supplementary information

## References

[1] Institute of Medicine. *Dietary Reference Intakes for Calcium and Vitamin D*. (National Academies Press (US), 2011). 21796828

[2] Chinese Nutrition Society. *Chinese Dietary Reference Intakes* (*2013*). (Science Press, 2014).

[3] Joint FAO/WHO Expert Consultation on Human Vitamin and Mineral Requirements. *Vitamin and mineral requirements in human nutrition: report of a joint FAO/WHO expert consultation*, *Bangkok*, *Thailand*. 2 edn, (World Health Organization, 2004).

[4] He Y, Zhai F, Wang Z, Hu Y (2007). Status of dietary calcium intake of Chinese residents. Wei Sheng Yan Jiu.

[5] Du WW, Wang HJ, Wang ZH, Zhai FY, Zhang B (2010). Trend of milk consumption among Chinese children and adolescents aged 7 to 17 years old in 9 provinces from 1991 to 2006. Zhonghua liu xing bing xue za zhi.

[6] Prentice A (2005). Calcium supplementation increases stature and bone mineral mass of 16- to 18-year-old boys. J Clin Endocrinol Metab.

[7] Dodiuk-Gad RP, Rozen GS, Rennert G, Rennert HS, Ish-Shalom S (2005). Sustained effect of short-term calcium supplementation on bone mass in adolescent girls with low calcium intake. Am J Clin Nutr.

[8] Dibba B (2000). Effect of calcium supplementation on bone mineral accretion in gambian children accustomed to a low-calcium diet. Am J Clin Nutr.

[9] Matkovic V (2005). Calcium supplementation and bone mineral density in females from childhood to young adulthood: a randomized controlled trial. Am J Clin Nutr.

[10] Zhu K (2006). Growth, bone mass, and vitamin D status of Chinese adolescent girls 3 y after withdrawal of milk supplementation. Am J Clin Nutr.

[11] Lee WT, Leung SS, Leung DM, Cheng JC (1996). A follow-up study on the effects of calcium-supplement withdrawal and puberty on bone acquisition of children. Am J Clin Nutr.

[12] Winzenberg, T. M., Shaw, K., Fryer, J. & Jones, G. Calcium supplementation for improving bone mineral density in children. *Cochrane Database Syst Rev*, CD005119, doi:10.1002/14651858.CD005119.pub2 (2006). 10.1002/14651858.CD005119.pub2 PMC8865374 16625624

[13] Winzenberg T, Shaw K, Fryer J, Jones G (2007). Calcium supplements in healthy children do not affect weight gain, height, or body composition. Obesity (Silver Spring).

[14] Zhang B, Zhai FY, Du SF, Popkin BM (2014). The China Health and Nutrition Survey, 1989-2011. Obes Rev.

[15] Wang Y, Ge K, Popkin BM (2000). Tracking of body mass index from childhood to adolescence: a 6-y follow-up study in China. Am J Clin Nutr.

[16] de Onis M (2007). Development of a WHO growth reference for school-aged children and adolescents. Bull World Health Organ.

[17] World Health Organization. *Child growth standards: Length/height-for-age*, http://www.who.int/childgrowth/standards/height_for_age/en/ (Date of access: 10/01/2016) (2007).

[18] Zhai F (1996). The evaluation of the 24-hour individual dietary recall method in China. Wei Sheng Yan Jiu.

[19] Institute of Health of the Chinese Academy of Medical Sciences. *Food Composition Table* (*1981*). (People’s Medical Publishing House, 1980).

[20] Institute for Nutrition and Food Hygiene of the Chinese Academy of Preventive Medicine. *Food Composition Table* (*1991*). (People’s Medical Publishing House, 1991).

[21] Institute for Nutrition and Food Safety of the Chinese Center for Disease Control and Prevention. *China Food Composition Table* (*2002*). (Peking University Medical Press, 2002).

[22] Institute for Nutrition and Food Safety of the Chinese Center for Disease Control and Prevention. *China Food Composition Table* (*2004*). (Peking University Medical Press, 2005).

[23] Willett WC, Howe GR, Kushi LH (1997). Adjustment for total energy intake in epidemiologic studies. Am J Clin Nutr.

[24] Jones-Smith JC, Popkin BM (2010). Understanding community context and adult health changes in China: development of an urbanicity scale. Soc Sci Med.

[25] Desquilbet L, Mariotti F (2010). Dose-response analyses using restricted cubic spline functions in public health research. Stat Med.

[26] Malina, R. M., Bouchard, C. & Bar-Or, O. *Growth*, *maturation*, *and physical activity*. 2nd edn, (Human Kinetics, 2004).

[27] Black RE, Williams SM, Jones IE, Goulding A (2002). Children who avoid drinking cow milk have low dietary calcium intakes and poor bone health. Am J Clin Nutr.

[28] Lee WT (1995). A randomized double-blind controlled calcium supplementation trial, and bone and height acquisition in children. Br J Nutr.

[29] Bonjour JP (1997). Calcium-enriched foods and bone mass growth in prepubertal girls: a randomized, double-blind, placebo-controlled trial. J Clin Invest.

[30] Bonjour JP, Chevalley T, Ammann P, Slosman D, Rizzoli R (2001). Gain in bone mineral mass in prepubertal girls 3.5 years after discontinuation of calcium supplementation: a follow-up study. Lancet.

[31] Chevalley T, Rizzoli R, Hans D, Ferrari S, Bonjour JP (2005). Interaction between calcium intake and menarcheal age on bone mass gain: an eight-year follow-up study from prepuberty to postmenarche. J Clin Endocrinol Metab.

[32] Iuliano-Burns S, Wang XF, Evans A, Bonjour JP, Seeman E (2006). Skeletal benefits from calcium supplementation are limited in children with calcium intakes near 800 mg daily. Osteoporos Int.

[33] Prentice A, Dibba B, Sawo Y, Cole TJ (2012). The effect of prepubertal calcium carbonate supplementation on the age of peak height velocity in Gambian adolescents. Am J Clin Nutr.

[34] Stear SJ, Prentice A, Jones SC, Cole TJ (2003). Effect of a calcium and exercise intervention on the bone mineral status of 16-18-y-old adolescent girls. Am J Clin Nutr.

[35] Zong XN, Li H (2013). Construction of a new growth references for China based on urban Chinese children: comparison with the WHO growth standards. PLoS One.

[36] Thomis MA, Towne B (2006). Genetic determinants of prepubertal and pubertal growth and development. Food Nutr Bull.

[37] Allen LH (1994). Nutritional influences on linear growth: a general review. Eur J Clin Nutr.

[38] Yin J (2010). Factors affecting calcium balance in Chinese adolescents. Bone.

[39] Vatanparast H, Bailey DA, Baxter-Jones AD, Whiting SJ (2010). Calcium requirements for bone growth in Canadian boys and girls during adolescence. Br J Nutr.

[40] Cannata D, Vijayakumar A, Fierz Y, LeRoith D (2010). The GH/IGF-1 axis in growth and development: new insights derived from animal models. Advances in pediatrics.

[41] Ginty, F. *et al*. In *Nutritional aspects of osteoporosis* (eds Burckhardt, P. Dawson-Hughes, B. & Heaney, R. P.) 45–57 (Elsevier Science (USA), 2004).

[42] Millward, D. J. Nutrition, infection and stunting: the roles of deficiencies of individual nutrients and foods, and of inflammation, as determinants of reduced linear growth of children. *Nutr Res Rev*, 1–23, doi:10.1017/s0954422416000238 (2017). 10.1017/S0954422416000238 28112064

[43] Nishiyama KK (2012). Cortical porosity is higher in boys compared with girls at the distal radius and distal tibia during pubertal growth: an HR-pQCT study. J Bone Miner Res.

[44] Schoenau E, Neu CM, Rauch F, Manz F (2002). Gender-specific pubertal changes in volumetric cortical bone mineral density at the proximal radius. Bone.

[45] Duan Y, Beck TJ, Wang XF, Seeman E (2003). Structural and biomechanical basis of sexual dimorphism in femoral neck fragility has its origins in growth and aging. J Bone Miner Res.

[46] Schiessl H, Frost HM, Jee WS (1998). Estrogen and bone-muscle strength and mass relationships. Bone.

[47] Zhu K (2008). Growth and bone mineral accretion during puberty in Chinese girls: a five-year longitudinal study. J Bone Miner Res.

