# Supplementary material for: Low Habitual Dietary Calcium and Linear Growth from Adolescence to Young Adulthood: results from the China Health and Nutrition Survey
Source: Sci Rep. 2017 Aug 22;7:9111. doi: 10.1038/s41598-017-08943-6 (PMC5567300; doi:10.1038/s41598-017-08943-6)
Supplement: Supplementary file 1 — Supplementary information [file 41598_2017_8943_MOESM1_ESM.pdf]

Low Habitual Dietary Calcium and Linear Growth from Adolescence to Young Adulthood: results from the China Health and Nutrition Survey

Aiping Fang,[1,2] Keji Li,[2] He Li,[2] Meihan Guo,[2] Jingjing He,[2] Xin Shen,[2] Jie Song[2]

Supplementary Information

Supplemental Tables

Supplemental Table S1. Baseline characteristics of the growth cohort before and after excluding ineligible participants

	Boys			Girls		
	Before (*n*=1539)	After (*n*=1165)	*P*	Before (*n*=1150)	After (*n*=854)	*P*
Age (years) [a]	15.5(2.3)	15.4(2.3)	0.2766	13.6(2.2)	13.5(2.3)	0.3645
Height (cm) [a]	157.8(12.0)	157.3(12.0)	0.2760	147.2(10.6)	146.7(10.8)	0.3238
Weight (kg) [a]	47.4(11.5)	47.0(11.4)	0.3156	39.4(9.4)	39.1(9.4)	0.5158
BMI (kg/m^2) [a]	18.8(2.6)	18.7(2.6)	0.3390	18.0(2.7)	17.9(2.7)	0.7306
HAZ[a]	-1.22(1.12)	-1.24(1.13)	0.6368	-1.17(1.10)	-1.18(1.11)	0.9445
Paternal height (cm) [a]	165.0(6.3)	165.2(6.3)	0.5171	165.4(6.4)	165.6(6.4)	0.5731
Maternal height (cm) [a]	154.4(6.0)	154.6(5.9)	0.3645	154.6(5.9)	154.9(6.0)	0.2609
Daily dietary intake[d]						
Total energy (kcal) [a]	2532(737)	2522(673)	0.7209	2065(563)	2061(534)	0.8678
Protein (g) [a]	74.9(25.2)	75.0(24.5)	0.9022	62.4(19.9)	62.2(19.1)	0.8648
Vitamin A (ugRE) [b]	341(173,615)	343(184,610)	0.8957	312(153,553)	291(146,528)	0.1484
Calcium (mg) [a]	417(220)	415(199)	0.8077	360(196)	349(166)	0.1778
Iron (mg) [a]	23.0(10.9)	23.3(11.7)	0.4899	19.3(8.3)	19.6(8.7)	0.4484
Zinc (mg) [a]	12.0(3.9)	12.0(3.6)	0.9816	9.9(3.1)	9.8(3.0)	0.7376
Urbanization index[a]	47.2(17.8)	47.9(17.7)	0.2921	48.4(18.3)	49.8(17.9)	0.0896
Latitude[a]	31.3(6.2)	31.3(6.2)	0.8593	31.9(6.2)	32.2(6.3)	0.1984
DNR (kWh/m^2/d) [a]	3.46(1.04)	3.47(1.06)	0.7538	3.57(1.05)	3.60(1.07)	0.4851
Year of birth[b]	1979(1975,1983)	1979(1975,1984)	0.6201	1980(1977,1985)	1981(1977,1986)	0.1667
Wave at entry[c]			0.7008			0.5615
1989-1993	883(59.4)	709(60.9)		697(62.2)	512(60.0)	
1997-2000	512(34.4)	390(33.5)		354(31.6)	289(33.8)	

	Boys			Girls		
	Before (*n*=1539)	After (*n*=1165)	*P*	Before (*n*=1150)	After (*n*=854)	*P*
2004-2009	92(6.2)	66(5.7)		70(6.2)	53(6.2)	
Stratum[c]			0.7697			0.7084
Rural	1166(78.4)	908(77.9)		847(75.6)	639(74.8)	
Urban	321(21.6)	257(22.1)		274(24.4)	215(25.2)	
Household income level[c]			0.9523			0.5364
Low	523(35.2)	408(35.0)		443(39.5)	319(37.4)	
Medium	651(43.8)	506(43.4)		466(41.6)	360(42.2)	
High	313(21.0)	251(21.5)		212(18.9)	175(20.5)	

[a]Data shown is mean(SD), and two-independent samples t tests were used to compare the characteristics before and after excluding ineligible participants.

[b]Data shown is median(IQR), and two-independent samples nonparametric tests were used to compare the characteristics before and after excluding ineligible participants.

[c]Data shown is *n*(%), and chi-square tests were used to compare the characteristics before and after excluding ineligible participants.

[d]Energy adjusted average nutrient intakes during adolescence were estimated from a mean number of 1.8 three-day 24-hour dietary recalls (range: 1-3) obtained during age 12-19 in boys and 10-17 years in girls using the residual method after log transformation.

BMI: body mass index; HAZ: height-for-age *z*-score; DNR: annual average direct normal radiation; IQR: interquartile range.

Supplemental Table S2. Mean daily food consumption during adolescence in Chinese boys and girls

	Boys (*n*=1165)	Girls (*n*=854)
Plant foods [a]		
Cereals (g/1000 kcal)	196.0(1.2)	191.8(1.4)
Vegetables (g/1000 kcal)	122.6(2.1)	129.3(2.6)
Tubers (g/1000 kcal)	14.9(1.0)	17.0(1.3)
Vegetable oil (g/1000 kcal)	9.1(0.2)	9.7(0.3)
Soybean (g/1000 kcal)	5.9(0.3)	6.4(0.3)
Fruit (g/1000 kcal)	5.3(0.5)	9.0(1.0)
Mixed beans (g/1000 kcal)	2.6(0.3)	2.2(0.3)
Nuts and seeds (g/1000 kcal)	0.7(0.1)	0.8(0.1)
Animal foods [a]		
Meat (g/1000 kcal)	24.6(0.7)	24.8(0.8)
Eggs (g/1000 kcal)	6.2(0.3)	7.7(0.4)
Seafood (g/1000 kcal)	5.9(0.3)	6.5(0.4)
Animal oil (g/1000 kcal)	4.0(0.2)	3.8(0.2)
Poultry (g/1000 kcal)	2.9(0.2)	2.8(0.3)
Milk (g/1000 kcal)	2.8(0.4)	3.3(0.6)

[a] Data shown is mean(SE).

Supplemental Table S3. Mean dietary protein and calcium from main food sources during adolescence in Chinese boys and girls

	Boys (*n*=1165)				Girls (*n*=854)			
	Protein		Calcium		Protein		Calcium	
	g/1000 kcal [a]	%	mg/1000 kcal [a]	%	g/1000 kcal [a]	%	mg/1000 kcal [a]	%
Plant foods	23.6 (0.2)	80.2	152.6 (1.8)	92.0	23.7 (0.2)	79.3	155.8 (2.1)	91.4
Cereals	17.7(0.1)	60.6	34.3(0.6)	23.3	17.4(0.2)	58.7	35.1(0.7)	23.1
Vegetables	1.9(0.03)	6.7	72.9(1.6)	42.5	2.1(0.04)	7.1	73.6(1.9)	41.6
Soybean	2.1(0.1)	6.4	23.1(0.9)	12.7	2.2(0.1)	6.9	25.2(1.2)	13.5
Mixed beans	0.6(0.1)	1.8	3.6(0.4)	2.0	0.5(0.1)	1.5	2.7(0.4)	1.6
Tubers	0.3(0.02)	0.9	2.1(0.2)	1.4	0.3(0.02)	1.0	2.1(0.2)	1.4
Animal foods	6.2 (0.2)	19.6	13.3 (0.7)	7.7	6.5 (0.2)	20.3	15.5 (1.0)	8.2
Meat	3.7(0.1)	11.9	1.8(0.1)	1.2	3.8(0.1)	11.9	1.8(0.1)	1.2
Seafood	1.0(0.1)	3.1	4.8(0.4)	2.7	1.1(0.1)	3.4	5.5(0.5)	2.8
Eggs	0.8(0.04)	2.5	3.4(0.2)	2.1	1.0(0.05)	3.2	4.1(0.2)	2.5
Poultry	0.5(0.04)	1.6	0.4(0.04)	0.3	0.5(0.1)	1.5	0.4(0.05)	0.2
Milk	0.1(0.01)	0.3	2.8(0.5)	1.3	0.1(0.02)	0.3	3.6(0.7)	1.4

[a] Data shown is mean(SE).

Supplemental Table S4. Distribution of percentage of mean dietary protein and calcium from plant foods during adolescence in Chinese boys and girls

	Percentage of the nutrient from plant foods [a]			
	<25%	25%~50%	50%-75%	≥75%
Boys				
Protein	0(0.0)	53(4.5)	378(32.5)	734(63.0)
Calcium	1(0.1)	16(1.4)	70(6.0)	1078(92.5)
Girls				
Protein	0(0.0)	49(5.7)	280(32.8)	525(61.5)
Calcium	0(0.0)	16(1.9)	53(6.2)	785(91.9)

[a] Data shown is *n*(%).

Supplemental Table S5. Linear regression analysis of energy and energy adjusted nutrient intakes and participants' age during adolescence in Chinese boys and girls

	B	*SE*	*t*	*P*	R^2	adjusted R^2
Boys						
Total energy	73.85	6.57	11.23	<.0001	0.0636	0.0631
Protein[a]	-0.06	0.14	-0.45	0.65	0.654	-0.0004
Vitamin A[a]	7.76	7.73	1.00	0.32	0.316	<0.0001
Calcium[a]	-0.37	1.86	-0.20	0.84	0.840	-0.0005
Iron[a]	0.08	0.10	0.84	0.40	0.399	-0.0002
Zinc[a]	0.03	0.02	1.39	0.17	0.166	0.0005
Girls						
Total energy	32.22	6.20	5.20	<.0001	<.0001	0.0181
Protein[a]	0.17	0.14	1.16	0.25	0.247	0.0002
Vitamin A[a]	-0.81	8.09	-0.10	0.92	0.921	-0.0007
Calcium[a]	1.86	1.88	0.99	0.32	0.324	<0.0001
Iron[a]	-0.02	0.08	-0.24	0.81	0.811	-0.0007
Zinc[a]	0.02	0.02	0.75	0.46	0.456	-0.0003

[a] Energy adjusted nutrient intakes were estimated with dietary records from each survey during adolescence using the residual method.
